# Bystander activation of irrelevant CD4^+^ T cells following antigen-specific vaccination occurs in the presence and absence of adjuvant

**DOI:** 10.1371/journal.pone.0177365

**Published:** 2017-05-10

**Authors:** Susan van Aalst, Irene S. Ludwig, Ruurd van der Zee, Willem van Eden, Femke Broere

**Affiliations:** Department of Infectious Diseases and Immunology, Utrecht University, Utrecht, The Netherlands; Weizmann Institute of Science, ISRAEL

## Abstract

Autoimmune and other chronic inflammatory diseases (AID) are prevalent diseases which can severely impact the quality of life of those that suffer from the disease. In most cases, the etiology of these conditions have remained unclear. Immune responses that take place e.g. during natural infection or after vaccination are often linked with the development or exacerbation of AID. It is highly debated if vaccines induce or aggravate AID and in particular adjuvants are mentioned as potential cause. Since vaccines are given on a large scale to healthy individuals but also to elderly and immunocompromised individuals, more research is warranted. Non-specific induction of naïve or memory autoreactive T cells via bystander activation is one of the proposed mechanisms of how vaccination might be involved in AID. During bystander activation, T cells unrelated to the antigen presented can be activated without (strong) T cell receptor (TCR) ligation, but via signals derived from the ongoing response directed against the vaccine-antigen or adjuvant at hand. In this study we have set up a TCR transgenic T cell transfer mouse model by which we were able to measure local bystander activation of transferred and labeled CD4^+^ T cells. Intramuscular injection with the highly immunogenic Complete Freund’s Adjuvant (CFA) led to local *in vivo* proliferation and activation of intravenously transferred CD4^+^ T cells in the iliac lymph node. This local bystander activation was also observed after CFA prime and Incomplete Freund’s Adjuvant (IFA) boost injection. Furthermore, we showed that an antigen specific response is sufficient for the induction of a bystander activation response and the general, immune stimulating effect of CFA or IFA does not appear to increase this effect. In other words, no evidence was obtained that adjuvation of antigen specific responses is essential for bystander activation.

## Introduction

The adaptive response of the immune system is antigen specific and therefore uniquely directed against the pathogen it is confronted with. In principle this occurs in the absence of responses against neighboring harmless environmental antigens or self-antigens. However, adaptive immune responses to antigens not included in the pathogen initially encountered were shown, known as heterologous reactions [1–4]. Through molecular mimicry, T cells that respond against an antigen in the pathogen presented (classical response), may cross react with an antigen that differs from the one initially presented (heterologous response). The heterologous response is thus executed by the same T cell that is involved in the classical response [5]. This is in contrast to another type of heterologous response; the one due to bystander activation. In bystander activation, the heterologous response is performed by an adjacent, non-relevant T cell with a specificity that is different from that involved in the classical response. The heterologous T cell is thought to be activated without (strong) TCR ligation, but via cytokines like IL-2 as result of the (excessive) activation of cells during the classical response [4,6,7].

During (viral) infections, bystander activation of CD8^+^ T cells is a well described phenomenon [8]. Bystander activation of both naïve [9] and memory CD8^+^ T cells [10–13] is reported, though it remains difficult to completely exclude the possibility of cross reactivity as underlying factor of this heterologous response. Bystander activation of CD4^+^ T cells is less well studied, but it was demonstrated that unrelated memory CD4^+^ T cells can be activated after a recall tetanus vaccination via bystander activation [14–16]. Furthermore, infection with *Leishmania donovani* affects heterologous memory as well as naïve CD4^+^ T cells [17].

The overall impact of infection-induced bystander activation is not yet completely clear. Although it might seem remarkable that the stringent antigen-specificity of the adaptive immune system can be circumvented, some hypothesized that the activation of surrounding memory T cells is actually beneficial for the immune system as it might maintain or strengthen the memory T cell repertoire [1,10,15,17]. On the other hand, bystander activation during natural infection might pose a risk as well. Non-specific induction of naïve or memory autoreactive T cells could potentially lead to the development of autoimmune disease (AID) or the induction of a relapse in the AID respectively.

Natural infection is often implicated in the onset or exacerbations of AID but the underlying involved mechanisms are mostly not known [2,7,18,19]. Similarly, vaccinations—simulating natural infections—may also be involved in the onset or exacerbations of AID [20–23], in which in particular adjuvants are suspected to be implicated. Shoenfeld raised awareness on adjuvants involved in AID and introduced the term ‘autoimmune/inflammatory syndrome induced by adjuvants’ (ASIA; [24]), which is since then a highly debated topic [25–27]. Importantly, though sufficient suspected individual cases have been reported, epidemiological studies do not substantiate evident causal relationships between vaccination and AID (reviewed in [28,29]). Despite several (mouse) studies [15,30,31], reviewed in [20], it is still highly debated if and how vaccinations induce or worsen AID. A number of mechanisms, amongst which bystander activation, are suggested [2,7,18,19,32]. Since vaccinations are given on a large scale to healthy adults but also to children, elderly and immunocompromised individuals, more research is warranted.

In this study, we set out to develop a method to test bystander activation of non-vaccine specific CD4^+^ T cells by adjuvants or vaccines. For this purpose we successfully set up a T cell receptor transgenic (TCR Tg) T cell transfer mouse-model by which we were able to measure bystander activation of such unrelated, CD4+ T cells after a prime or prime-boost immunization with the highly immune stimulating and reactogenic adjuvant Complete Freund’s adjuvant (CFA; [33]) or with antigen alone without adjuvant. In the prime-boost immunization both prime and boost led to bystander activation. Interestingly, bystander activation was also seen when antigen was administered in the absence of adjuvant. Therefore, it appeared that the antigen-specific CD4^+^ T cell expansion and activation, and not the adjuvant-mediated stimulation during vaccination is essential for the bystander response.

## Material and methods

### Ethical statement

All animal experiments were performed in strict accordance to the Dutch Animal Experimentation Act and EU directives 86/609/CEE and 2010/63/EU related to the protection of vertebrate animals used for experimental and other scientific purposes and were approved by the Committee on Animal Experiments of the University of Utrecht (DEC2014.II.02.008) and performed in the Central Laboratory Animal Research Facility of the University of Utrecht (GDL), which has AAALAC (Association for Assessment and Accreditation of Laboratory Animal Care) accreditation.

### Animals

BALB/c mice (male, 8–10 weeks) were purchased from Charles River Laboratories. Human proteoglycan (hPG) specific TCR-5/4E8-transgenic (Tg) mice [34] were bred at the GDL under specified pathogen free conditions. To introduce the congenic marker Thy1.1 (CD90.1), TCR-5/4E8-Tg mice were back-crossed on CBy.PL(B6)-Thy1a/ScrJ mice (purchased from The Jackson Laboratory). Male and female offspring with the Thy1.1 phenotype (Thy1.1^+^TCR-5/4E8-Tg; >8 weeks) were used in experiments. During experiments, mice were kept under standard conditions and received water and food *ad libitum*. Mice were randomly divided in control- or treatment groups and all animals were monitored on the first two day post (each) immunization and from thereon weekly. No signs of pain or other distress were observed.

### *In vivo* transfer studies

Single cell suspensions from spleens of TCR-5/4E8-Tg or Thy1.1^+^TCR-5/4E8-Tg donor mice were CD4^+^ T cell enriched and labeled with 5,6-carboxy-succinimidyl-fluoresceine-ester (CFSE) as described before [35]. In short, splenocytes were incubated with monoclonal antibodies specific for CD45R (Ra3-6B2), MHC class II (M5/114), CD11b (M1/70) and CD8 (YTS169) and positive cells were removed with sheep-anti-rat conjugated Dynal beads (Invitrogen). The remaining, enriched CD4^+^ T cells (purity routinely between 85–95%), were labeled for 10 min with 0.5 μM CFSE and suspended in PBS.

**Fig 1:**
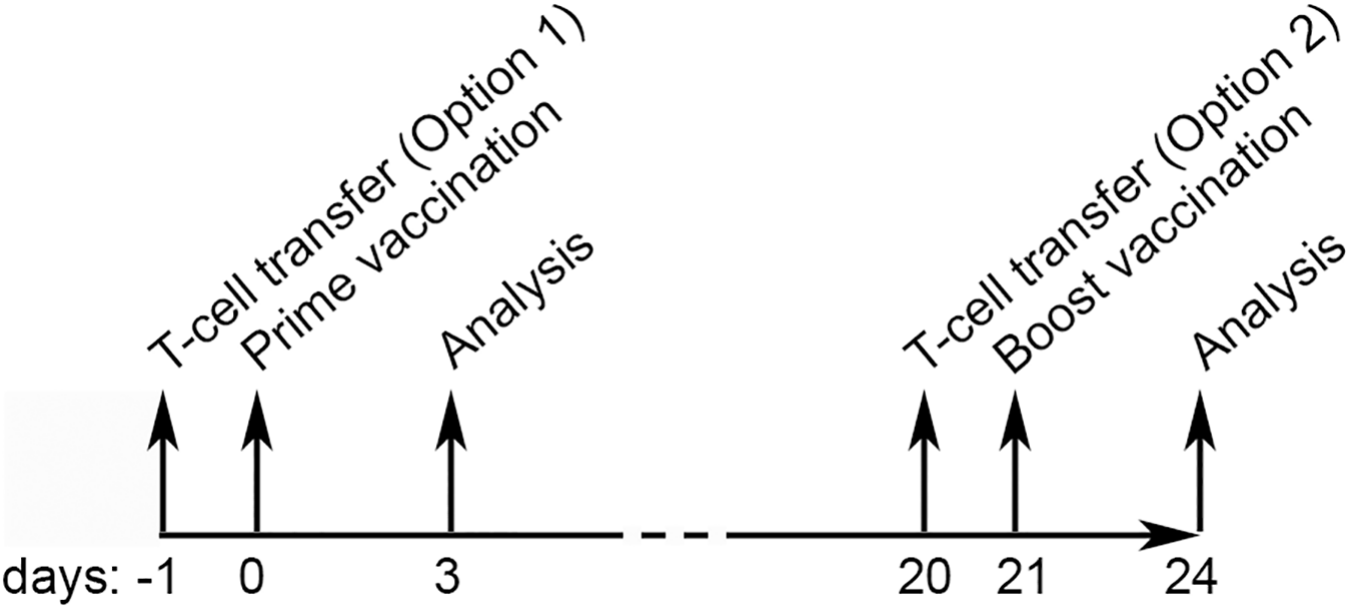
Set up of *in vivo* transfer studies. CD4^+^ T cells were isolated from TCR Tg mice which have CD4^+^ T cells specific for hPG peptide. The (CD90.1^+^)CD4^+^ T cells were CFSE-labeled and i.v. transferred to donor mice at d_-1_ (approach 1) or d_20_ (approach 2). At d_0_ acceptor mice received an i.m. injection (50 μl) in the left quadriceps with CFA or PBS, sometimes supplemented with hPG-peptide or OVA. Animals were sacrificed at d_3_ or d_24_. Animals sacrificed on d_24_ received a booster shot (right quadriceps, d_21_) with IFA or PBS, sometimes supplemented with OVA.

Acceptor WT Balb/c mice received intravenously (i.v.) 3x10^6^ CFSE-labeled CD4^+^ T cells in 200 μl PBS at the indicated time point (d_-1_/d_20_; Fig 1). At d_0_ mice were intramuscularly (i.m.) primed (left quadriceps) with 50 μl Complete Freund’s Adjuvant (CFA; Difco, 1 mg/ml *M*. *butyricum;* 1:1 mixed with PBS) or PBS (Lonza) ± antigen. At d_21_ mice were boosted (i.m.; right quadriceps) with 50 μl Incomplete Freund’s Adjuvant (IFA; Sigma; 1:1 mixed with PBS) or PBS ± antigen. Antigens were human proteoglycan peptide (hPG; 100μg, ^70^ATEGRVRVNSAYQDK^84^; Genscript) or Ovalbumin (OVA; 100μg; grade 7; Sigma). At d_3_ or d_24_ acceptor mice were euthanized using a carbon dioxide chamber and spleens and draining iliac lymph node, inguinal lymph node and non-draining brachial lymph node (ndLN) were isolated.

### Single cell suspensions

Single cell suspensions from spleens and LN were prepared using 70 μm cell strainers (BD Bioscience). Erythrocytes were removed in ACK-lysis buffer (150 mM NH_4_CL, 1 mM NaHCO_3_; pH 7.40). Cells were suspended in PBS supplemented with 2% FBS (Lonza) for flow cytometric staining or in IMDM (Gibco) containing FBS (5%), β-mercaptoethanol (Gibco; 5x10^-5^ M), penicillin (Gibco; 100 units/ml) and streptomycin (Gibco; 100 μg/ml) (complete medium) for culturing.

### Re-stimulation-assay and ELISpot

In the re-stimulation assay, single cell suspensions of spleen and LN of immunized acceptor mice were cultured in 200 μl complete medium for 24 hours in 96-wells round bottom plates (Corning) at 1x10^6^ cells/well in the presence of 20 μg/ml hPG peptide or medium. After culture, cells were used for flow cytometric analysis.

The number of IFN-y producing cells was determined in an IFN-y ELISpot. Multiscreen IP Filter Plate plates (Millipore) were activated with 70% Ethanol, coated with a rat anti-mouse anti-IFN-y antibody (clone AN-18, homemade) at 2 μg/ml in PBS and then blocked with IMDM medium supplemented with 5% FBS. Subsequently, single cell suspensions of spleens and LN of acceptor mice were cultured at 5x10^5^ cells/well in 200 μl complete medium for 48 hours in the presence of 20 μg/ml hPG peptide, 100 μg/ml OVA or medium. Then, plates were washed and the IFN-γ producing cells were detected using the rat anti-mouse biotin-anti-IFN-y antibody (homemade, clone XMG1.2) and streptavidin-alkaline phosphatase (Sigma, S2890). Spots were visualized using BCIP/NPT solution (Roche) according to manufacturer’s instructions. Counting of the IFN-γ producing cells was done by the Automated ELISpot Assay Video Analysis System (A.EL.VIS GmbH).

### Flow cytometry

Cells were stained with a combination of rat anti-mouse monoclonal antibodies CD4-PE (H129.19, IgG2a, BD Biosciences), CD4-eFluor450 (RM4-5, IgG2a, BD Biosciences), CD62L-PE (MEL-14, IgG2a, BD Biosciences), CD62L-BV510 (MEL-14, IgG2a, BD Biosciences), Armenian-Hamster anti-mouse CD69-BV510 (H1.2F3, IgG, BD Biosciences) and CD69-APC (H1.2F3, eBioscience) and mouse-anti-mouse/rat Thy1.1-PerCPCy5.5 (HIS51, IgG2a, eBioscience). Subsequently, cells were measured on a FACSCanto II Flow cytometer (BDBiosciences). Analysis was performed with FlowJo v7.6.5 (Tree Star). Proliferation of the transferred cells was defined as % divided CFSE^+^ (CD90.1^+^)CD4^+^ T cells in the live cell gate.

### Statistical analysis

Statistical analysis was performed using Prism 6 v6.05 (GraphPad). Differences between two groups were determined with an unpaired two-tailed student’s t-test. Differences between three groups were determined with a one-way ANOVA (two-tailed) with Dunnett’s multiple comparison test. P < 0.05 was considered significant.

## Results

To test the possible induction of bystander activation by (adjuvanted) vaccines, we have set up a TCR Tg T cell-transfer mouse model. In this model, acceptor mice receive CFSE-labeled hPG-specific CD4^+^ T cells and activation and/or proliferation of these vaccine-unrelated cells after a prime/prime-boost immunization is the read out of bystander activation. In our experiments both the effect of prime and boost were considered (Fig 1).

### Intramuscular injection with hPG peptide induces proliferation and activation of transferred hPG-specific CD4^+^ T cells

**Fig 2:**
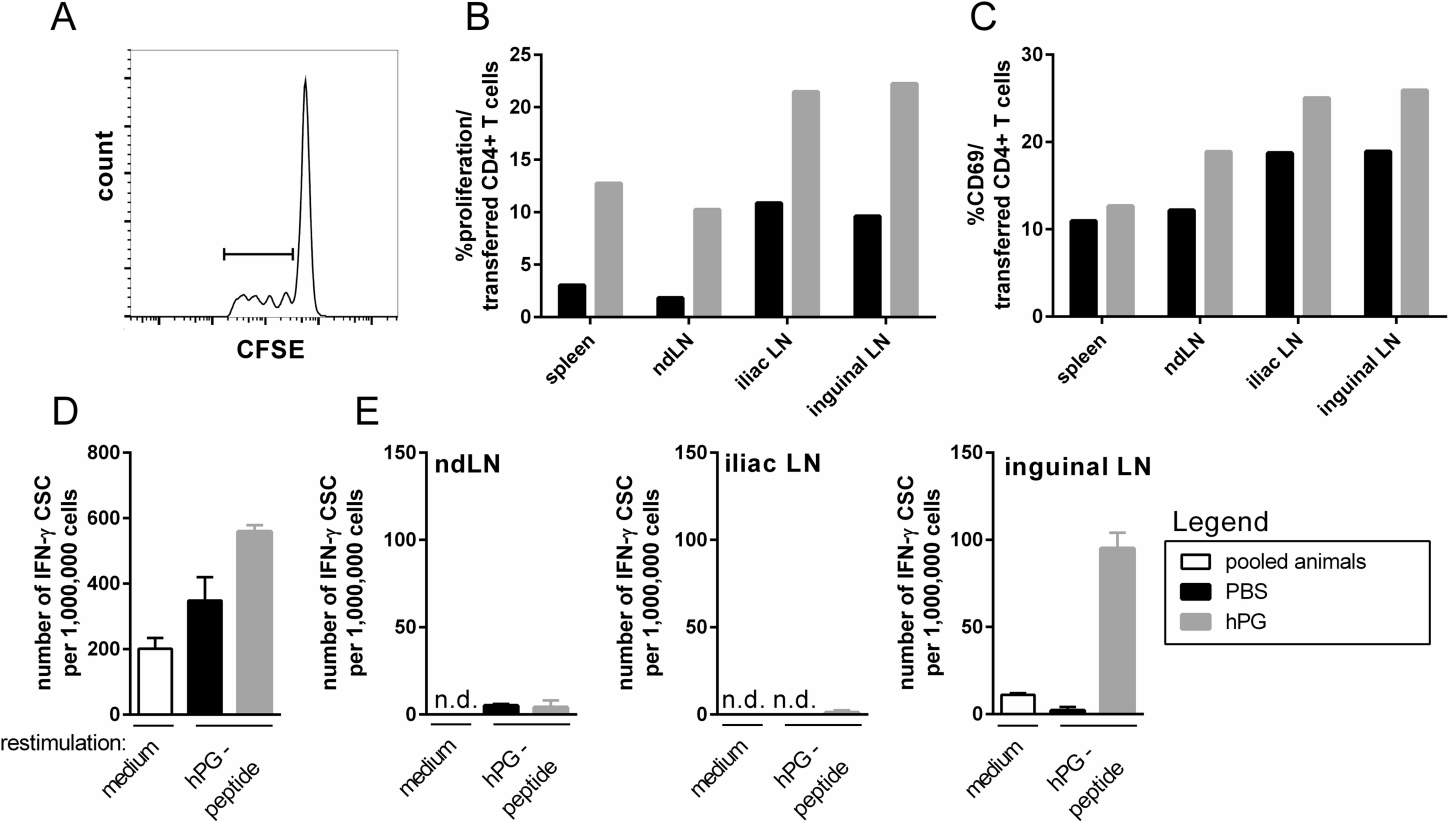
Injection with hPG peptide results in proliferation and activation of transferred hPG-T cells at d_3_. Animals received an i.v. transfer of CFSE-labeled hPG specific CD4^+^ T cells at d_-1_ and an i.m. injection with PBS or hPG peptide in PBS at d_0_ before analysis at d_3_. (A) Representative FACS-histogram and -gating strategy of CFSE-labeled hPG-specific donor CD4^+^ T cells in the iliac LN after hPG peptide injection. (B,C) Percentage of (B) proliferation of and (C) CD69 expression on CFSE-labeled CD4^+^ T cells in spleen and LN. (D,E) IFN-γ-ELISpot assay with 48h *ex vivo* hPG peptide restimulation of (D) splenocytes and (E) LNs. The ELISpot was performed in duplo (LN) and triplo (spleen). 1 animal per group was used. This experiment was performed twice. ND: not determined, hPG: hPG peptide, CSC: cytokine secreting cell, med: medium, ndLN: non-draining LN.

Before we considered the bystander effect of immunization, we confirmed that an i.m. injection with hPG peptide, the cognate antigen of the transferred CD4^+^ T cells, induced activation of the transferred T cells. We observed increased proliferation of the transferred cells in the draining iliac and inguinal LN, the non-draining brachial LN (ndLN) as well as in spleen compared to PBS (Fig 2A and 2B). Furthermore, a higher % of the transferred CD4^+^ T cells in the hPG-injected acceptor expressed the activation marker CD69 (Fig 2C) and, after 48h *in vitro* restimulation with hPG peptide, more IFN-γ producing cells were observed in the spleen (Fig 2D) and inguinal LN, but not in the iliac LN or ndLN after hPG peptide injection (Fig 2E).

### CFA prime and CFA/IFA prime-boost immunization induce local bystander activation

**Fig 3:**
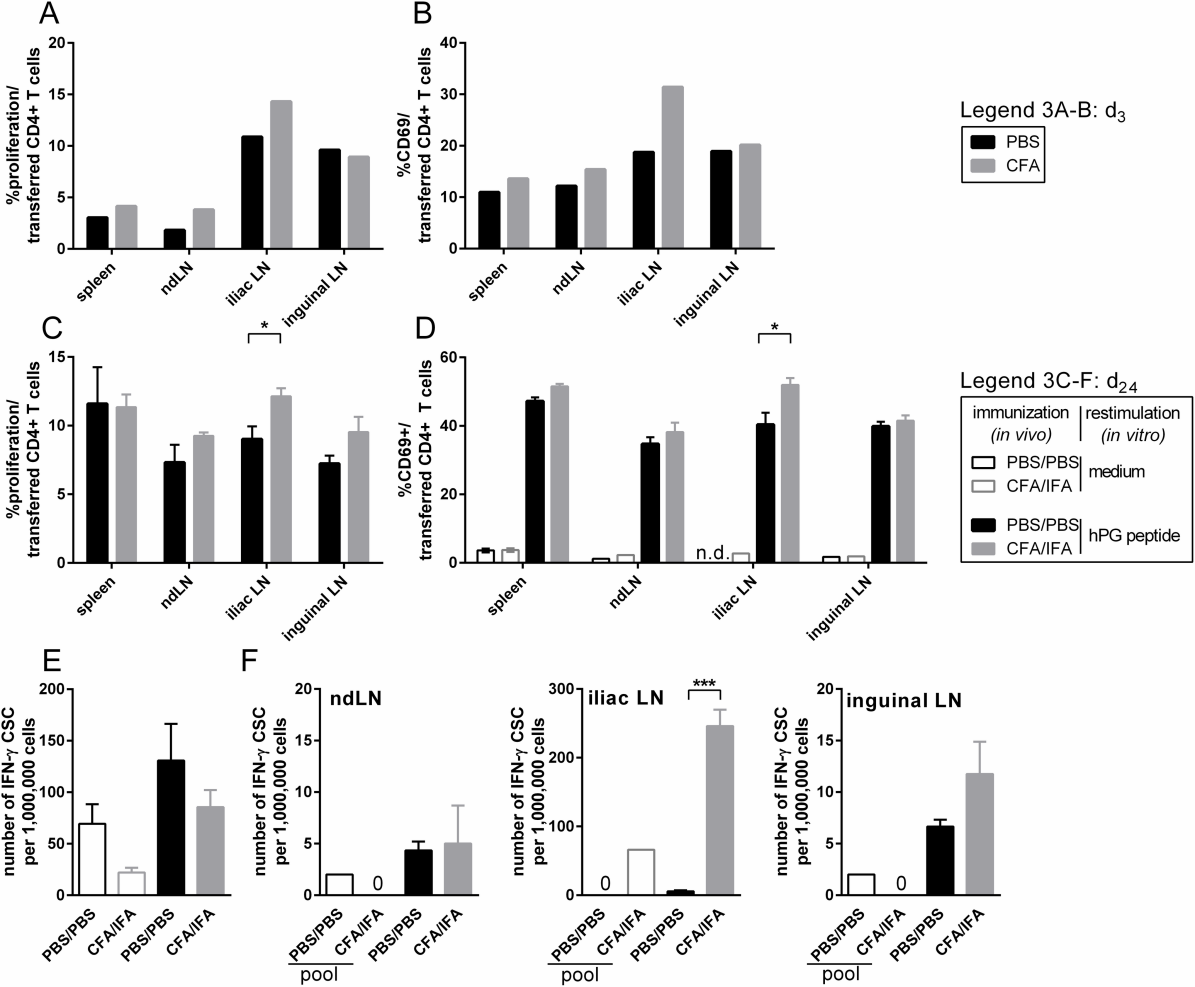
CFA prime and CFA/IFA prime-boost leads to local activation and proliferation of transferred hPG-T cells. (A, B) Animals received an i.v. transfer of CFSE-labeled hPG specific CD4^+^ T cells at d_-1_ and an i.m. injection with PBS or CFA at d_0_ before analysis at d_3_. Percentage of (A) proliferation of and (B) CD69 expressing cells in CFSE-labeled CD4^+^ cell population in spleen and LN. (C-F) Animals received an i.v. transfer of CFSE-labeled hPG specific CD4^+^CD90.1^+^ T cells at d_-1_ and an i.m. prime with PBS or CFA at d_0_, followed by a boost with PBS or IFA at d_21_. (C,D) At d_24_ cells were restimulated with hPG peptide for 24h. Indicated are the percentage of (C) proliferation of and (D) CD69 expressing cells in CFSE-labeled CD90.1^+^CD4^+^ cells in spleen and LN after *ex vivo* hPG restimulation. (E,F) At d_24_ (E) splenocytes and (F) LNs were used for an IFN-y-ELISpot assay with 48h *ex vivo* hPG-peptide restimulation. The ELISpot was performed in duplo (LN) and triplo (spleen).Results were obtained with N = 3–4. In some cases, LN cells were pooled per group for the medium stimulus (pool). Means+SEM are shown. Differences between two groups were determined with an unpaired two-tailed student’s t-test. P < 0.05 was considered significant. ND: not determined, CSC: cytokine secreting cell, ndLN: non-draining LN.

To investigate the effect of a prime (d_0_) immunization with CFA on bystander activation, we analyzed the effect of CFA immunization on the transferred CD4^+^ T cells at d_3_ in a pilot experiment (transfer at d_-1_; Fig 3A and 3B). The transferred CD4^+^ T cells present in the iliac LN after CFA immunization appeared to have proliferated more than in a control animal (Fig 3A) and were more activated as measured by their CD69 expression (Fig 3B). This effect was not observed in the inguinal LN (Fig 3A and 3B). Furthermore, contrary to what was observed after hPG-injection (Fig 2B), CFA-injection did not induce obvious systemic proliferation or activation of the transferred CD4^+^ T cells in the spleen or ndLN (Fig 3A and 3B) at this time point.

Subsequently, we considered the effect of a CFA prime (d_0_) plus IFA boost (d_21_) on the transferred CD4^+^ T cells (transfer at d_-1_; Fig 3C–3F). Three days after the IFA boost (d_24_) and 24h *ex vivo* restimulation with hPG peptide, a significantly increased proliferation of transferred CD4^+^ T cells in the iliac LN was observed after CFA/IFA immunization compared to PBS. A similar trend could also be seen in the inguinal and ndLN (Fig 3C). *Ex vivo* hPG peptide restimulation of the CD4^+^ T cells led to upregulation of the CD69 activation marker compared to medium stimulation only in both PBS and CFA/IFA injected animals, however, in the iliac LN hPG peptide restimulated cells of the CFA/IFA immunization showed significantly higher CD69 expression than after PBS (Fig 3D). Similarly, more IFN-γ producing cells were found in the iliac LN after CFA/IFA compared to PBS, but not in the inguinal LN, ndLN or spleen (Fig 3E and 3F).

Thus, in particular locally, the transferred CFA non-related CD4^+^ T cells were activated and able to proliferate as a result of CFA prime or CFA/IFA prime-boost injection, showing local bystander activation of surrounding CD4^+^ T cells.

### Antigen-specific boost induces local bystander activation

In Fig 3 we showed bystander activation of transferred hPG-specific CD4^+^ T cells both after CFA-prime and after CFA/IFA-prime-boost immunization. However, in that set up, the immediate effect of boost could not directly be determined. Furthermore, IFA does not contain antigens and thus an IFA immunization does not lead to an actual adaptive boost response. Therefore we tested the effect of an antigen-specific boost by supplementing OVA to all PBS and CFA/IFA injections and changed the time of CD4^+^ T cell transfer to 1 day before boost (d_20_; approach 2 in Fig 1). Analysis was performed 3 days post boost at d_24_.

**Fig 4:**
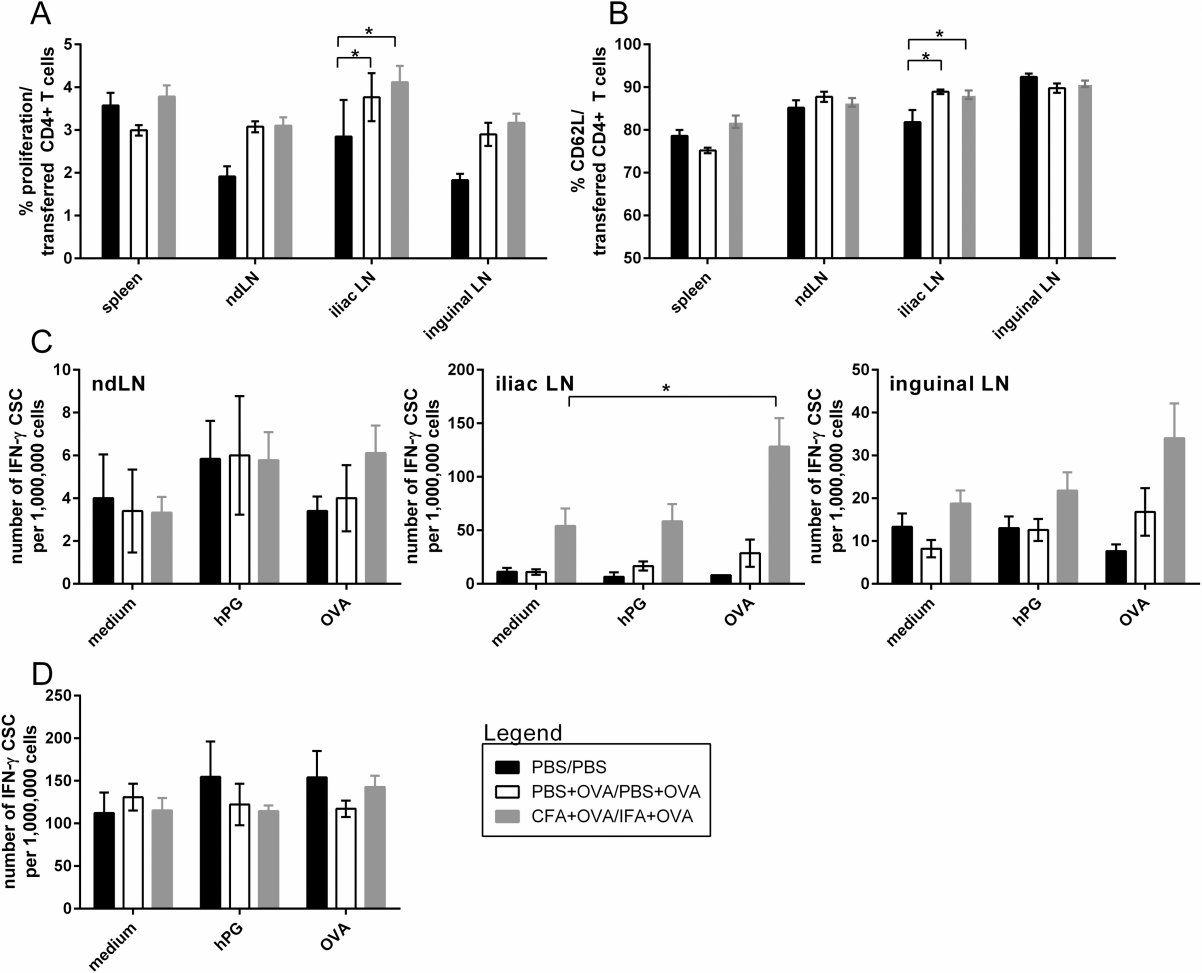
Antigen-specific boost leads to local proliferation of transferred hPG-specific T cells. Animals received a prime at d_0_ (PBS, OVA+PBS or OVA+CFA) followed by an i.v. transfer of labeled CD90.1^+^CD4^+^ T cells at d_20_ and a boost at d_21_ (PBS, OVA+PBS or OVA+IFA) before analysis at d_24_. (A,B) Percentage of (A) proliferation of and (B) CD62L expression in CFSE-labeled CD90.1^+^CD4^+^ cells in spleen and LN. (C,D) IFN-γ-ELISpot assay with 48h *ex vivo* hPG-peptide restimulation of (D) splenocytes and (C) LNs. The ELISpot was performed in duplo (LN) and triplo (spleen). Data shown are the means+SEM of two independent experiments (each 2–5 animals per group). Differences between two groups were determined with an unpaired two-tailed student’s t-test. Differences between the three groups were determined with a one-way ANOVA (two-tailed) with Dunnett’s multiple comparison test. P < 0.05 was considered significant. CSC: cytokine secreting cell, ndLN: non-draining LN.

Proliferation of transferred CD4^+^ T cells was increased after both OVA+PBS and OVA+CFA/IFA immunizations compared to PBS, in the inguinal LN (significant), ndLN (significant) and iliac LN (trend) but not in spleen. However, no differences in proliferation were observed between the two OVA immunizations (Fig 4A). In most organs, transferred CD4^+^ T cells expressed similar amounts of CD62L (mainly expressed on naïve T cells) regardless of the type of immunization, only in the iliac LN CD62L expression was higher for both OVA immunizations compared to PBS (Fig 4B).

Compared to medium, *in vitro* hPG-peptide restimulation did not lead to an increased number of IFN-γ producing cells after either OVA-immunization (Fig 4C and 4D) in LNs or spleen. Unrelated to the *in vitro* antigenic stimulus, we did observe more IFN-γ producing cells after OVA+CFA/IFA compared to OVA+PBS or PBS alone in the iliac and inguinal LN (Fig 4D), which was also observed after CFA immunization without OVA (Fig 3F, iliac LN). Both OVA immunizations induced IFN-γ producing cells in the draining LN that were OVA specific, since *ex vivo* OVA-restimulation resulted in an increase in IFN-γ producing cells compared to medium control. This effect was much stronger for OVA+CFA/IFA than OVA+PBS (Fig 4D). In general no systemic effects on IFN-γ producing cells could be observed in spleen or ndLN.

In sum, antigen-specific boost immunization contributes to the effect of bystander activation of the unrelated transferred CD4^+^ T cells, though the effect is only visible in the proliferation and not in the expression of activation markers or IFN-γ production.

## Discussion

Strong immune activation, such as during natural infection or after vaccines, is sometimes linked with the development or exacerbation of AID. Bystander activation of auto-reactive, non-vaccine related CD4^+^ T cells is one of the proposed mechanisms for this. In this study we have set up a TCR Tg T cell transfer mouse model (Figs 1 and 2) by which we were able to measure local bystander activation of CD4^+^ T cells after a CFA prime or CFA/IFA prime-boost injection (Fig 3). By supplementing the injections with the model-antigen OVA we showed that both prime and boost injections contribute to the effect and that not (only) the general immune stimulating effect of the CFA adjuvant, but also antigen-specific responses appear to contribute to bystander activation (Fig 4).

To certify that our transferred CD4^+^ T cells were able to be activated by an i.m. injection, we injected mice with their cognate peptide hPG, which was found to induce proliferation and activation of the transferred CD4^+^ T cells, both locally and systemically at d_3_ (Fig 2). IFN-γ production after hPG injection, as measured by ELISpot, was only visible in the inguinal LN, not iliac LN. This in contrast to what we found after CFA/IFA(±OVA) injections, where the iliac LN proved to be the most affected draining LN. No effects of injections were observed systemically in any of the conditions tested (Figs 3 and 4).

The experimental adjuvant CFA is known to induce strong inflammatory responses [33,36] including the induction of autoimmune arthritis in rats [37,38] and mice [39], and therefore not suitable for human vaccines [33]. It was included as the compound to secure maximal immune activation and indeed we were able to show that unrelated transferred CD4^+^ T cells were activated and proliferating after CFA prime injection as well as after CFA/IFA prime-boost injection (Fig 3) indicating bystander activation. The effect of the booster injection was not immediately clear from this experiment since the boost, IFA, does not induce such a pro-inflammatory milieu as CFA, nor does it contain antigenic compounds such as in CFA. Therefore, to investigate the effect of antigenic boost we included OVA in our prime-boost strategy and compared PBS injections with OVA+PBS and OVA+CFA/IFA prime-boost injections. To specifically test the effect of boost, we transferred the non-vaccine related CD4^+^ T cells at d_20_, just one day prior to boost (Fig 1, approach 2; Fig 4). Enhanced proliferation of the transferred CD4^+^ T cells as a consequence of antigenic boost was indeed visible after the OVA+CFA/IFA prime-boost injections compared to PBS, though effects were only observed in proliferation (Fig 4A) and not in the IFN-γ ELISpot (Fig 4C and 4D). This in contrast with the initial CFA prime-boost without OVA-supplementation where bystander activation was besides in proliferation (Fig 3A), also evident in the IFN-γ ELISpot (Fig 3D). Possibly, the transferred CD4^+^ T cells require to undergo both prime and boost before bystander activation can be detected by IFN-γ ELISpot, or the OVA-specific response interferes. As consequence of the influx and/or proliferation of OVA-specific cells, relatively lower number of transferred CD4^+^ T cells are present in the (iliac) LN (S1 Fig), resulting in lower numbers of hPG-specific transferred CD4^+^ T cells in the ELISpot assay and thus the bystander activation might be below the detection limit of the assay.

Remarkably, not only for OVA+CFA/IFA but also for OVA+PBS prime-boost we observed bystander activation (Fig 4A), suggesting that the boosting of an antigen-specific response is sufficient to induce bystander activation of surrounding CD4^+^ T cells and a highly immune stimulating adjuvant like CFA is not required nor significantly enhances the effect. We could exclude the possibility of cross-reactivity of our hPG-specific CD4^+^ T cells with OVA and/or mycobacterial antigens in CFA (S1 Table). Some proliferation was observed when hPG-specific CD4^+^ T cells were cultured with mycobacterial antigens, but this was also observed with other T cells (polyclonal or other transgenic specificity) and thus potentially an example of *in vitro* cytokine induced bystander activation via the production of IL-2 to which the hPG-specific CD4^+^ T cells were shown to respond (S2 Table). We thus show bystander activation as a result of an antigen-specific response in accordance with other reports [14,15] but which does not fit the ASIA hypothesis, which states that adjuvants and not (vaccine-)antigens lead to inflammatory diseases via a myriad of potential mechanisms, amongst which bystander activation [24,31]. The fact that the safe OVA+PBS prime-boost injection already induced bystander activation of CD4^+^ T cells could be a cause of concern; possibly it might be more frequent than anticipated and therefore could pose a risk on the induction of AID. However, even after years of (epidemiological) research, clear evidence of vaccine-induced AID is not yet established and likely the immune system has multiple check points before bystander induced AID is actually manifesting [40]. For example, the activation of naïve T cells via cytokines like lL-2, might not lead to functionally active CD4^+^ T cells, since this activation can also lead to apoptosis of these cells [41] and phenotypic activation (CD69 upregulation) not necessarily means cells will acquire effector function [42–44]. In line with this, we did not find IFN-γ production by bystander activated cells during the OVA prime-boost injections (Fig 4C and 4D). Thus, priming of circulating naïve self-reactive CD4^+^ T cells not necessarily leads to activated functional self-reactive CD4^+^ T cells. Furthermore, though we have shown that during a vaccine-antigen specific response, neighboring potentially self-reactive CD4^+^ T cells can be primed (Fig 4), the full activation of the self-reactive CD4^+^ T cells and subsequent development of AID, requires that the T cells recognize self-antigen in an inflamed surrounding at the tissue specific site, usually not the site of injection [45–48].

In this study, we have focused on the possibility of bystander activation of naïve T cells. We were able to show the bystander activation of CD4^+^ T cells from naïve donor animals, but the increase in proliferation and activation remained limited and local. Others found no [14,15] or limited effect [17] on bystander activation of naïve CD4^+^ T cells, but showed bystander activation of memory CD4^+^ T cells, similar to what was shown in infection for CD8^+^ T cells and which was mostly attributed to maintenance of memory [1,10,14–17]. Potentially, bystander activation of naïve and memory T cells could be occurring in parallel, and performing distinct functions and/or introducing different risks or even unexpected advantages in the case of memory cells. Off-target responses to vaccination are also found to be positive; vaccines bacille Calmette-Guérin and measles were found, amongst other to reduce all-cause mortality via heterologous responses, amongst which bystander is one of the proposed mechanism [49,50].

In conclusion, we have successfully set up a method to test bystander activation of non-vaccine specific CD4^+^ T cells by adjuvants or vaccines and were able to show local bystander activation after a prime-boost vaccination. Both prime and boost contribute to the effect and in particular the antigen, more than the adjuvant, appears to be responsible for the induction of bystander activation of CD4^+^ T cells.

## Supporting information

S1 Fig Total cells and frequency of CD90.1^+^CD4^+^ cells in LN after PBS±OVA or OVA+CFA/IFA prime-boost (d^24^). Animals received a prime at d_0_ (PBS, OVA+PBS or OVA+CFA) followed by an i.v. transfer of labeled CD4^+^CD90.1^+^ T cells at d_20_ and then a boost at d_21_ (PBS, OVA+PBS or OVA+IFA) before analysis at d_24_. Indicated are (A) the total numbers of cells and (B) the frequency of CD90.1^+^CD4^+^ cells in the ndLN, the iliac LN and the inguinal LN. Data shown are the mean of two independent experiments (each 2–5 animals per group). Each symbol represents an individual animal. Differences between two groups were determined with an unpaired two-tailed student’s t-test. P < 0.05 was considered significant.Black circles: PBS animals, light grey circles: OVA+PBS animals, dark grey squares: OVA+CFA animals.
(TIF)

S1 Table No cross-reactivity of hPG-specific CD4^+^ T cells with OVA or mycobacterial-antigens. Splenocytes from a TCR-5/4E8-Tg mouse, a mB29b-TCR Tg mouse [51] and Balb/c WT mouse were cultured in 200 μl complete medium for 72h at 2x10^5^ cells/well in the presence of 2 and 20 μg/ml OVA protein, H37Ra (*M*. *tuberculosis*), hPG peptide or B29-peptide, 2.5 μg/ml ConcavalinA or medium. Cells were stained with rat-anti-mouse antibodies CD4-APC (RM4-5; BD Biosciences), CD25-PerCPCy5.5 (PC61.5; eBioscience) and mouse-anti-human Ki67-PE (B56, BD Biosciences). Depicted is the delta percentage of Ki67+ cells in CD4^+^ cells after restimulation (percentage Ki67^+^CD4^+^ cellsrestimulation—percentage Ki67^+^CD4^+^ cells_medium_). Subsequently, cells were measured on a FACSCanto II Flow cytometer (BDBiosciences). Analysis was performed with FlowJo v7.6.5 (Tree Star).
(PDF)

S2 Table IL-2 induced hPG-specific CD4^+^ T cell proliferation. Splenocytes from a TCR-5/4E8-Tg mouse, a mB29b-TCR Tg mouse [51] and Balb/c WT mouse were cultured in 200 μl complete medium for 72h at 2x10^5^ cells/well in the presence of 20 and 100 U/ml IL-2 or medium. Cells were stained and analyzed as described in the legend of S1 Table.
(PDF)
