# Supplementary figures and images for: Bystander activation of irrelevant CD4+ T cells following antigen-specific vaccination occurs in the presence and absence of adjuvant
Source: PLoS One. 2017 May 10;12(5):e0177365. doi: 10.1371/journal.pone.0177365 (PMC5425230; doi:10.1371/journal.pone.0177365)

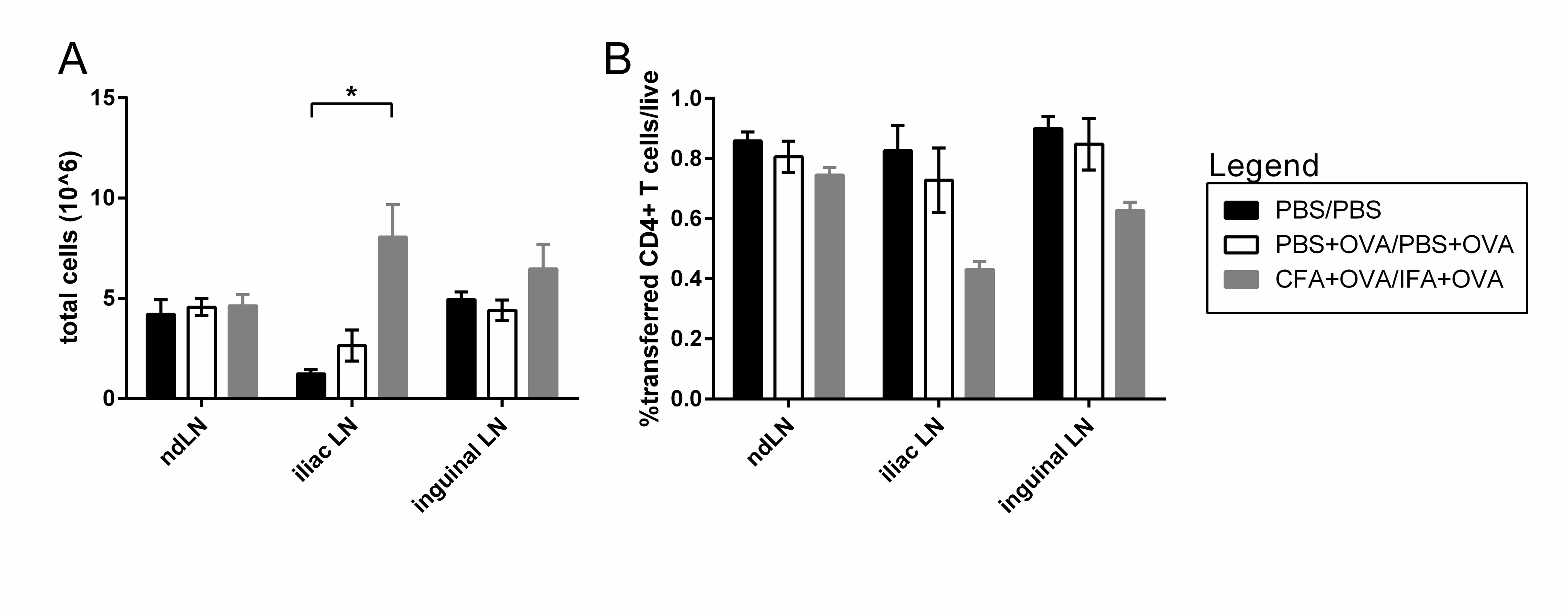

Supplement: S1 Fig — Animals received a prime at d0 (PBS, OVA+PBS or OVA+CFA) followed by an i.v. transfer of labeled CD4+CD90.1+ T cells at d20 and then a boost at d21 (PBS, OVA+PBS or OVA+IFA) before analysis at d24. Indicated are (A) the total numbers of cells and (B) the frequency of CD90.1+CD4+ cells in the ndLN, the iliac LN and the inguinal LN. Data shown are the mean of two independent experiments (each 2–5 animals per group). Each symbol represents an individual animal. Differences between two groups were determined with an unpaired two-tailed student’s t-test. P < 0.05 was considered significant.Black circles: PBS animals, light grey circles: OVA+PBS animals, dark grey squares: OVA+CFA animals. (TIF) [file pone.0177365.s001.tif]
