# Supplementary material for: Bystander activation of irrelevant CD4+ T cells following antigen-specific vaccination occurs in the presence and absence of adjuvant
Source: PLoS One. 2017 May 10;12(5):e0177365. doi: 10.1371/journal.pone.0177365 (PMC5425230; doi:10.1371/journal.pone.0177365)
Supplement: S1 Table — Splenocytes from a TCR-5/4E8-Tg mouse, a mB29b-TCR Tg mouse [51] and Balb/c WT mouse were cultured in 200 μl complete medium for 72h at 2x10^5^ cells/well in the presence of 2 and 20 μg/ml OVA protein, H37Ra (M. tuberculosis), hPG peptide or B29-peptide, 2.5 μg/ml ConcavalinA or medium. Cells were stained with rat-anti-mouse antibodies CD4-APC (RM4-5; BD Biosciences), CD25-PerCPCy5.5 (PC61.5; eBioscience) and mouse-anti-human Ki67-PE (B56, BD Biosciences). Depicted is the delta percentage of Ki67+ cells in CD4+ cells after restimulation (percentage Ki67+CD4+ cellsrestimulation—percentage Ki67+CD4+ cellsmedium). Subsequently, cells were measured on a FACSCanto II Flow cytometer (BDBiosciences). Analysis was performed with FlowJo v7.6.5 (Tree Star). (PDF) [file pone.0177365.s002.pdf]

Table S1 No cross-reactivity of hPG-specific $CD4^{+}$ T cells with OVA or mycobacterial-antigens

Δ%Ki67 expression (medium substracted)									
	OVA		Mtb		conA	hPG peptide		B29 peptide	
	2 µg/ml	20µg/ml	2 µg/ml	20µg/ml	2.5 µg/ml	2 µg/ml	20µg/ml	2 µg/ml	20µg/ml
TCR-5/4E8	0	1	2	7	68	73	75	0	2
mB29b-TCR	0	0	3	7	70	0	0	78	43
Balb/c WT	2	4	4	8	48	1	0	2	4
