# Supplementary material for: Bystander activation of irrelevant CD4+ T cells following antigen-specific vaccination occurs in the presence and absence of adjuvant
Source: PLoS One. 2017 May 10;12(5):e0177365. doi: 10.1371/journal.pone.0177365 (PMC5425230; doi:10.1371/journal.pone.0177365)
Supplement: S2 Table — Splenocytes from a TCR-5/4E8-Tg mouse, a mB29b-TCR Tg mouse [51] and Balb/c WT mouse were cultured in 200 μl complete medium for 72h at 2x10^5^ cells/well in the presence of 20 and 100 U/ml IL-2 or medium. Cells were stained and analyzed as described in the legend of S1 Table. (PDF) [file pone.0177365.s003.pdf]

Table S2 IL-2 induced hPG-specific $CD4^+$ T cell proliferation

Δ%Ki67 expression (medium substracted)		
	IL2	
	20U/ml	100U/ml
TCR-5/4E8	7	4
mB29b-TCR	7	3
Balb/c WT	11	10
